# Supplementary material for: An RNA-seq based comparative approach reveals the transcriptome-wide interplay between 3′-to-5′ exoRNases and RNase Y
Source: Nat Commun. 2020 Mar 27;11:1587. doi: 10.1038/s41467-020-15387-6 (PMC7101322; doi:10.1038/s41467-020-15387-6)
Supplement: Supplementary file 1 — Supplementary Information [file 41467_2020_15387_MOESM1_ESM.pdf]

SUPPLEMENTARY INFORMATION

An RNA-seq based comparative approach reveals the transcriptome-wide interplay between 3′-to-5′ exoRNases and RNase Y

Laura Broglia [1,2,3†], Anne-Laure Lécrivain [1,2,4†], Thibaud T. Renault [1,2,3], Karin Hahnke [1,2], Rina Ahmed-Begrich [1,2], Anaïs Le Rhun [1,2*] and Emmanuelle Charpentier [1,2,3,4*]

[1]Max Planck Unit for the Science of Pathogens, D-10117 Berlin, Germany

[2]Max Planck Institute for Infection Biology, Department of Regulation in Infection Biology, D-10117 Berlin, Germany

[3]Institute for Biology, Humboldt University, D-10115 Berlin, Germany

[4]The Laboratory for Molecular Infection Medicine Sweden (MIMS), Umeå Centre for Microbial Research (UCMR), Department of Molecular Biology, Umeå University, S-90187 Umeå, Sweden

[†]These authors contributed equally to this work.

*To whom correspondence should be addressed.

Tel: +33 55 7574 565; Email: anais.le-rhun@inserm.fr

Tel: +49 30 2846 0410; Fax: +49 30 2846 0412; Email: research-charpentier@mpusp.mpg.de

Keywords:

RNA degradation, RNA decay, ribonucleases, post-transcriptional regulation, RNase Y, YhaM, CBF1, PNPase, RNase R, RNA sequencing, Gram-positive bacteria, *Streptococcus pyogenes*.

SUPPLEMENTARY FIGURES

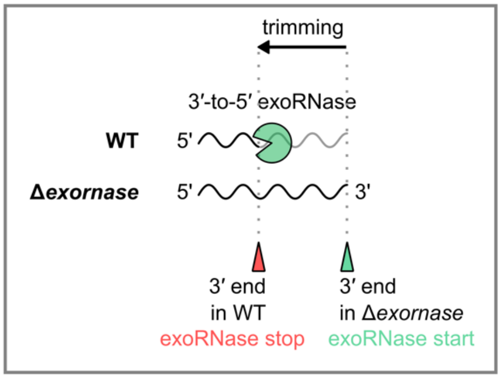

Supplementary Figure 1. Nomenclature of 3′-to-5′ exoRNase trimming positions.

The 3′-to-5′ exoRNase (‘pacman’ symbol) trims the RNA 3′ end from the start position (*i.e*. the end detected in Δ*exornase*, green arrow head) to the trimming stop position (*i.e*. the end detected in the WT, red arrowhead)[1].

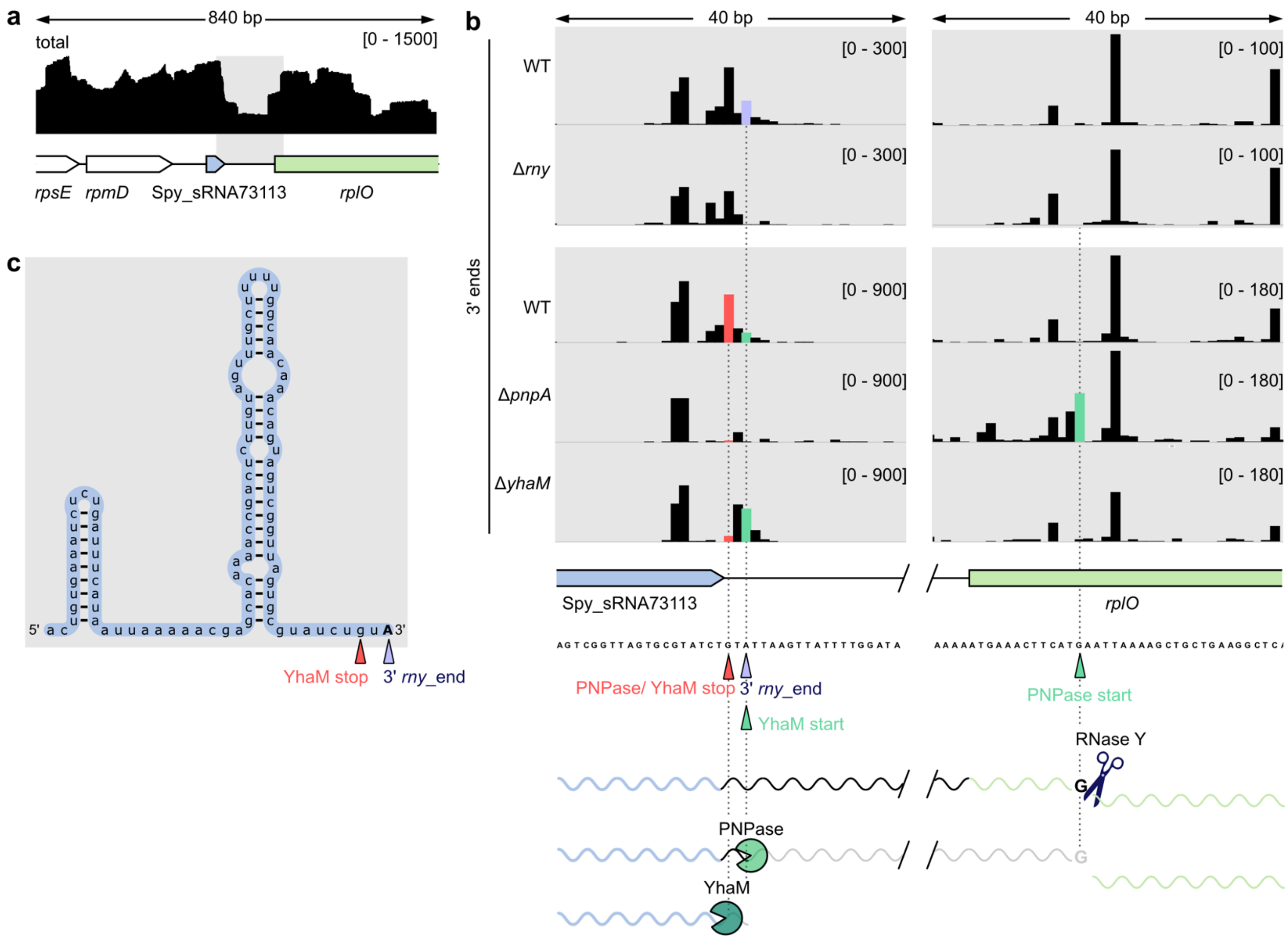

Supplementary Figure 2. Example of interplay between RNase Y and 3′-to-5′ exoRNases in the Spy_sRNA73113 <-> *rplO* intergenic region.

a Total coverage profiles in the WT strain obtained by RNA sequencing of the intergenic region between Spy_sRNA73113 (putative sRNA) and *rplO* coding for 50S ribosomal protein L15 (Spy_sRNA73113 <-> *rplO*) and schematic representation of the locus. The coverage scales are indicated between brackets. The grey rectangles indicate the regions where the processing sites of RNase Y, PNPase, and YhaM were identified. **b** 3′ end coverage of Spy_sRNA73113 <-> *rplO* in the WT, Δ*rny*, YhaM deletion mutant (Δ*yhaM*) and PNPase deletion mutant (Δ*pnpA*) strains. The scale for each lane is indicated between brackets. The detected 3′ *rny*_ends, the exoRNase trimming start and stop positions are depicted with purple, green and red arrow heads, respectively. The RNA was processed by RNase Y after a G, corresponding to the detected PNPase trimming start position. PNPase trimmed 120 nt of the Spy_sRNA73113 <-> *rplO* RNA 3′ end. This new RNA 3′ end was subsequently nibbled by YhaM. **c** RNA folding of the region 100 nt upstream of the 3′ *rny*_ends corresponding to YhaM trimming stop positions. YhaM started trimming after PNPase stopped, at the base of a stem loop structure, and consequently removed 2 nt from the RNA 3′ end.

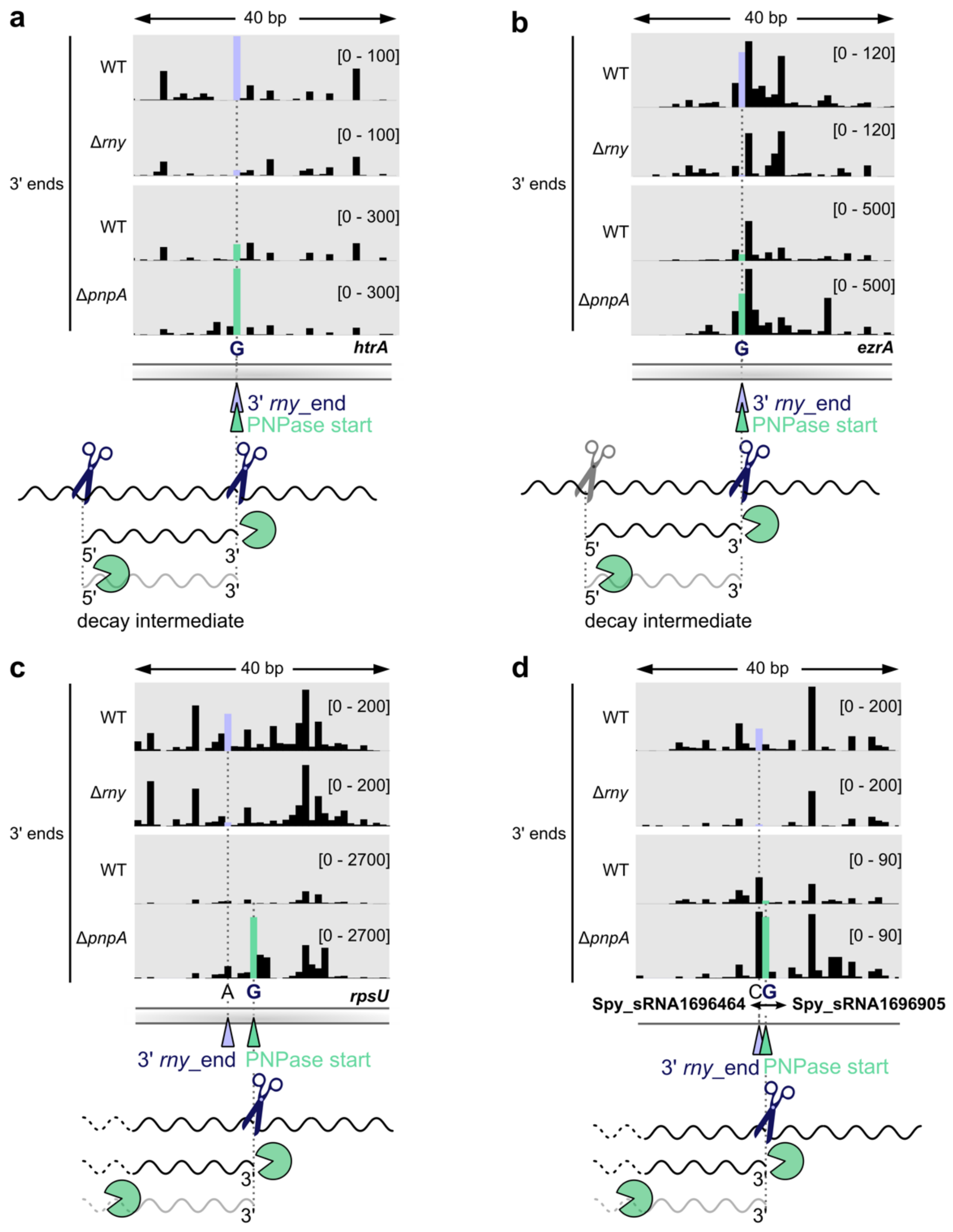

Supplementary Figure 3. RNA 3′ ends generated by RNase Y and targeted by PNPase.

a–d Examples of RNAs identified by matching the 3′ *rny*_ends (purple arrow heads) with PNPase trimming start positions (green arrowheads), as described in Figure 4 (Supplementary Data 4). For each RNA, the RNA 3′ end coverage profiles in the WT, Δ*rny*, and Δ*pnpA* strains, obtained by RNA sequencing are shown and the scales are indicated between brackets. Both RNase Y processing positions and PNPase trimming start sites were detected, indicating that all RNA 3′ ends generated by RNase Y (blue scissors) were targeted by PNPase ('pacman' symbol). **a, b *htrA* and *ezrA*.** Most of the RNA 3′ ends generated by RNase Y and eventually targeted by PNPase were part of decay intermediates. In some cases, RNase Y was also responsible for the generation of the decay intermediate 5′ ends (a). Alternatively, another endoRNase (grey scissors) produced the decay intermediate 5′ end, which was previously identified as RNA 5′ end more abundant in the Δ*pnpA* strain than in the WT strain[1] (b). **c, d *rpsU* and intergenic region between Spy_sRNA1696464 and**

Spy_sRNA1696905. The 3′ *rny*_ends matching the PNPase trimming start positions do not correspond to the 3′ ends of decay intermediates. As the PNPase trimming stop positions were not identified for these RNAs, it is likely that PNPase degrades up to the RNA termini.

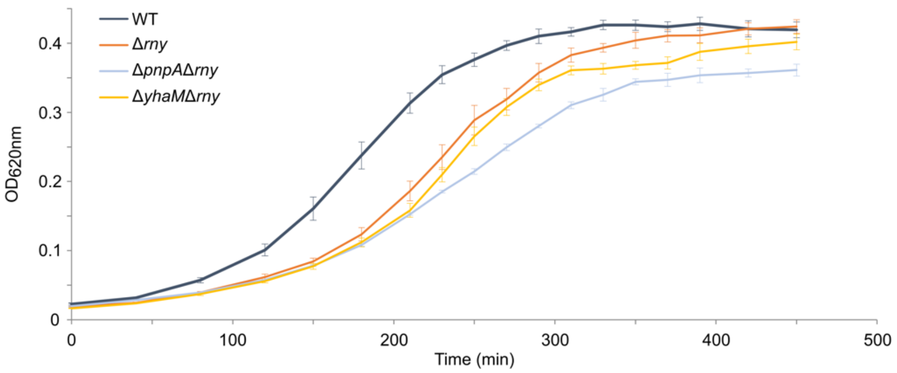

Supplementary Figure 4. The PNPase-RNase Y double deletion mutant has a stronger growth defect compared to the RNase Y single deletion mutant.

The WT, Δ*rny*, YhaM-RNase Y double deletion mutant (Δ*yhaM*Δ*rny*), and PNPase-RNase Y (Δ*pnpA*Δ*rny*) strains were grown in THY at 37°C with 5% CO_2 in triplicates and the optical density (OD) was monitored at 620_{nm} at regular time intervals. The standard error of the mean (s.e.m.) is indicated with error bars. Source data are provided as a Source Data file.

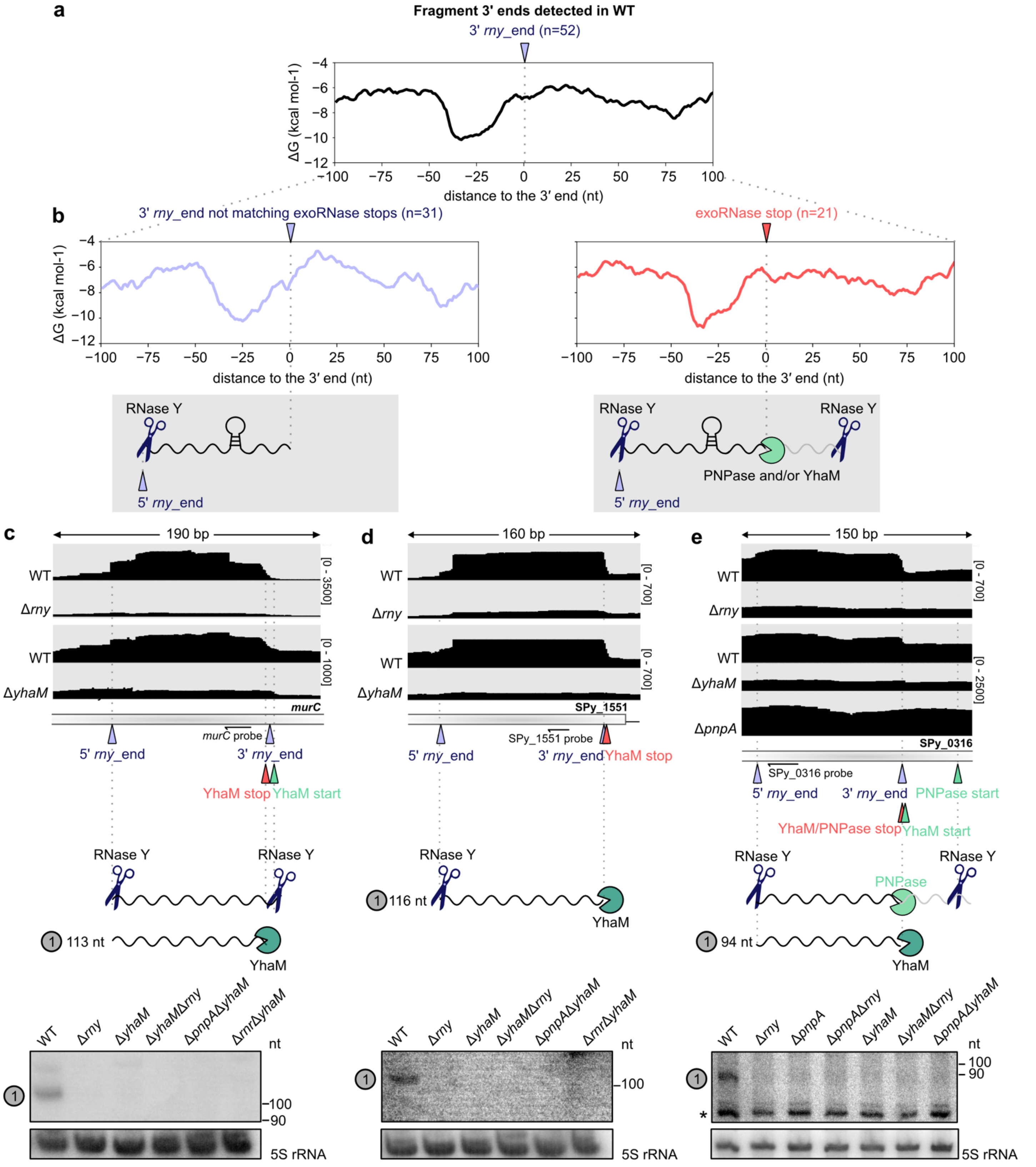

Supplementary Figure 5. Analysis of RNase Y-generated RNA fragments in WT.

a, b Structure conservation analysis surrounding the RNA fragment 3′ ends that are RNase Y-dependent. The average minimum free energy (ΔG in kcal mol[-1]) is shown for a region spanning 200

nt and centered on the 3′ ends of (a) total fragments, (b, left) fragments that were not identified as targets of 3′-to-5′ exoRNases, and (b, right) fragments trimmed by 3′-to-5′ exoRNases. A decrease in the ∆G that could indicate the presence of an RNA structure was observed in all the subgroups of the fragments analyzed. Schematic representations of the fragment generation and trimming are shown below the structure conservation analysis (panel b). RNase Y (blue scissors) generates both the 5′ and 3′ fragment ends. The fragment 3′ ends were in some cases subsequently trimmed by PNPase and/or YhaM (‘pacman’ symbol) from the start (green arrowhead) until the stop position (red arrowhead).
c–e Example of fragments produced by RNase Y and trimmed by 3′-to-5′ exoRNases. Top: total coverage profiles in the WT, ∆*rny*, and ∆*yhaM* strains (for the fragment in SPy_0316, also in the ∆*pnpA* strain) of fragments produced by RNase Y (scissors) and further trimmed by 3′-to-5′ exoRNases (‘pacman’ symbol) (see also Figure 6). The coverage scale, which is equal for lanes within the grey rectangles, is shown between brackets. The 3′ and 5′ *rny*_ends (purple arrowheads), the 3′-to-5′ exoRNase start and stop positions (green and red arrowheads) and the probes (arrows) used in the Northern blot analyses are indicated. When the 3′-to-5′ exoRNase trimming start position was retrieved, we identified the initial RNase Y (scissors) processing that generated the fragment 3′ end. Bottom: three RNA fragments (in c *murC*, d SPy_1551 and e SPy_0316) were analyzed by Northern blot performed in the WT, ∆*rny*, ∆*yhaM*, ∆*yhaM*∆*rny*, ∆*pnpA*∆*yhaM* and ∆*rnr*∆*yhaM* strains (for the fragment in SPy_0316, also in the ∆*pnpA* and ∆*pnpA*∆*rny* strains). The fragments were visible in the WT strain but not in the single and double RNase deletion strains. For the fragment in SPy_1551, which is poorly detectable, a contrasted portion of the blot is shown below the respective full blot. The star on the left of the Northern blot analysis for SPy_0316 indicates an RNA not specifically targeted by the probe. Shown are the results of one Northern blot analysis (n=3). The 5S rRNA was used as a loading control. Source data are provided as a Source Data file.

a

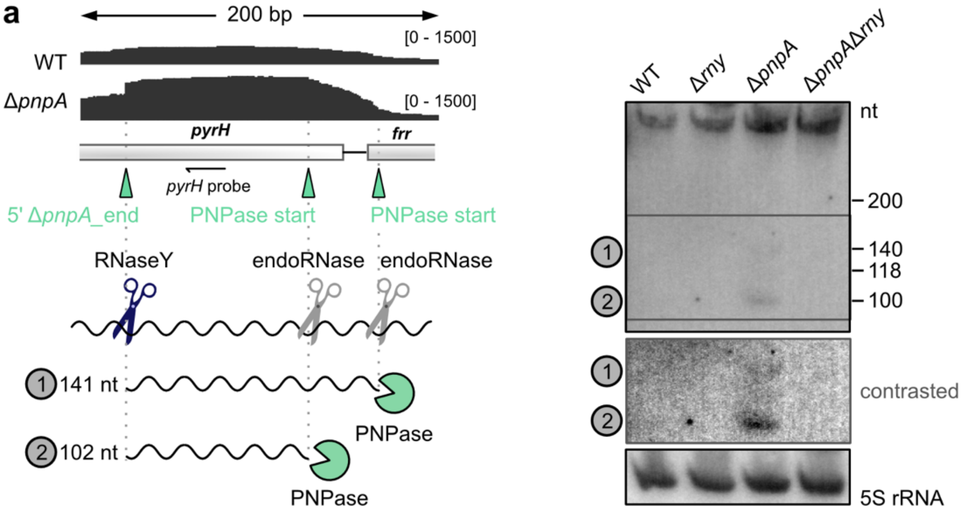

b

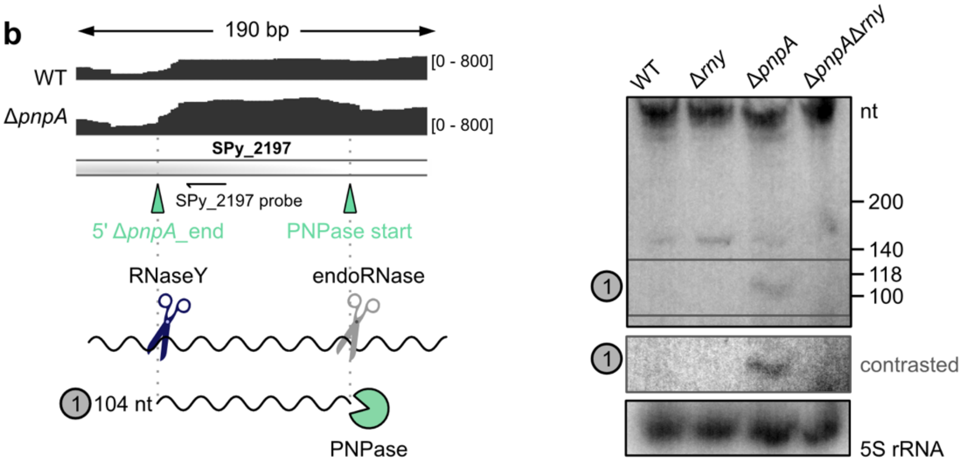

Supplementary Figure 6. RNase Y produces decay intermediates degraded by PNPase.

a, b Left: total coverage profiles of decay intermediates produced by RNase Y (blue scissors) and immediately degraded by PNPase ('pacman' symbol) in the WT and Δ*pnpA* strains (with scales between brackets) (see also Figure 7). The PNPase trimming start positions and the RNA 5′ ends that were more abundant in the Δ*pnpA* strain than the WT strain are depicted with green arrowheads. The predicted sizes of the decay intermediates are shown. Right: The Northern blot analyses were performed in the WT, Δ*rny*, Δ*pnpA,* and Δ*pnpA*Δ*rny* strains, and a contrasted portion of the blots is shown below the respective full blot. Shown are the results of one Northern blot analysis (n=3). The 5S rRNA was used as a loading control. Source data are provided as a Source Data file.

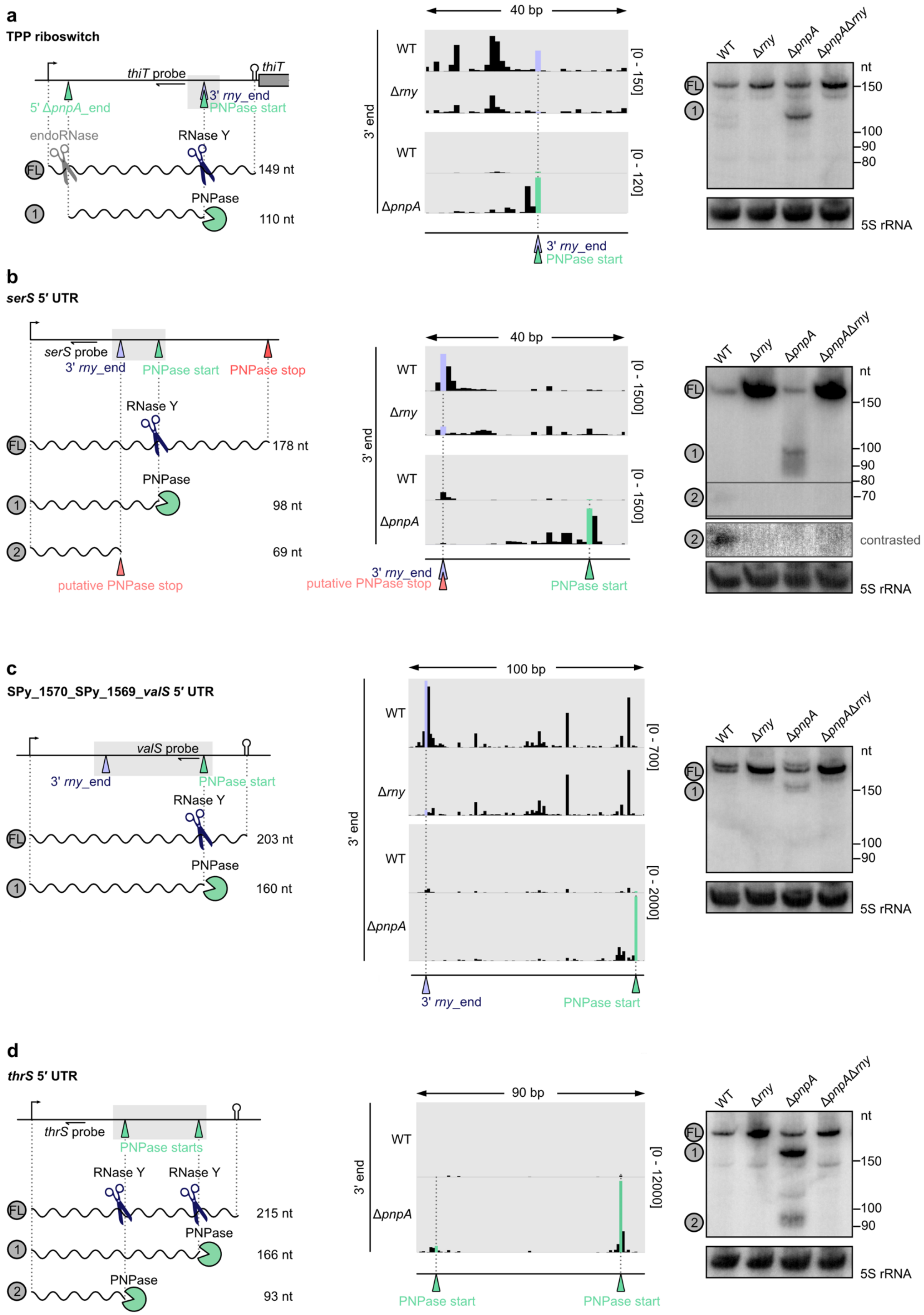

a
TPP riboswitch
thiT probe
thiT
5' ΔpnpA_end
3' rny_end
PNPase start
endoRNase
RNase Y
FL
149 nt
1
PNPase
110 nt
40 bp
3' end
WT
Δrny
ΔpnpA
[0 - 150]
[0 - 120]
WT
Δrny
ΔpnpA
ΔpnpAΔrny
nt
150
100
90
80
5S rRNA
b
serS 5' UTR
serS probe
3' rny_end
PNPase start
PNPase stop
RNase Y
FL
178 nt
1
PNPase
98 nt
2
69 nt
putative PNPase stop
40 bp
[0 - 1500]
nt
150
100
90
80
70
contrasted
5S rRNA
c
SPy_1570_SPy_1569_valS 5' UTR
valS probe
3' rny_end
PNPase start
RNase Y
FL
203 nt
PNPase
1
160 nt
100 bp
[0 - 700]
[0 - 2000]
nt
150
100
90
5S rRNA
d
thrS 5' UTR
thrS probe
PNPase starts
RNase Y
RNase Y
FL
215 nt
PNPase
1
166 nt
PNPase
2
93 nt
90 bp
[0 - 12000]
PNPase start
PNPase start
nt
150
100
90
5S rRNA

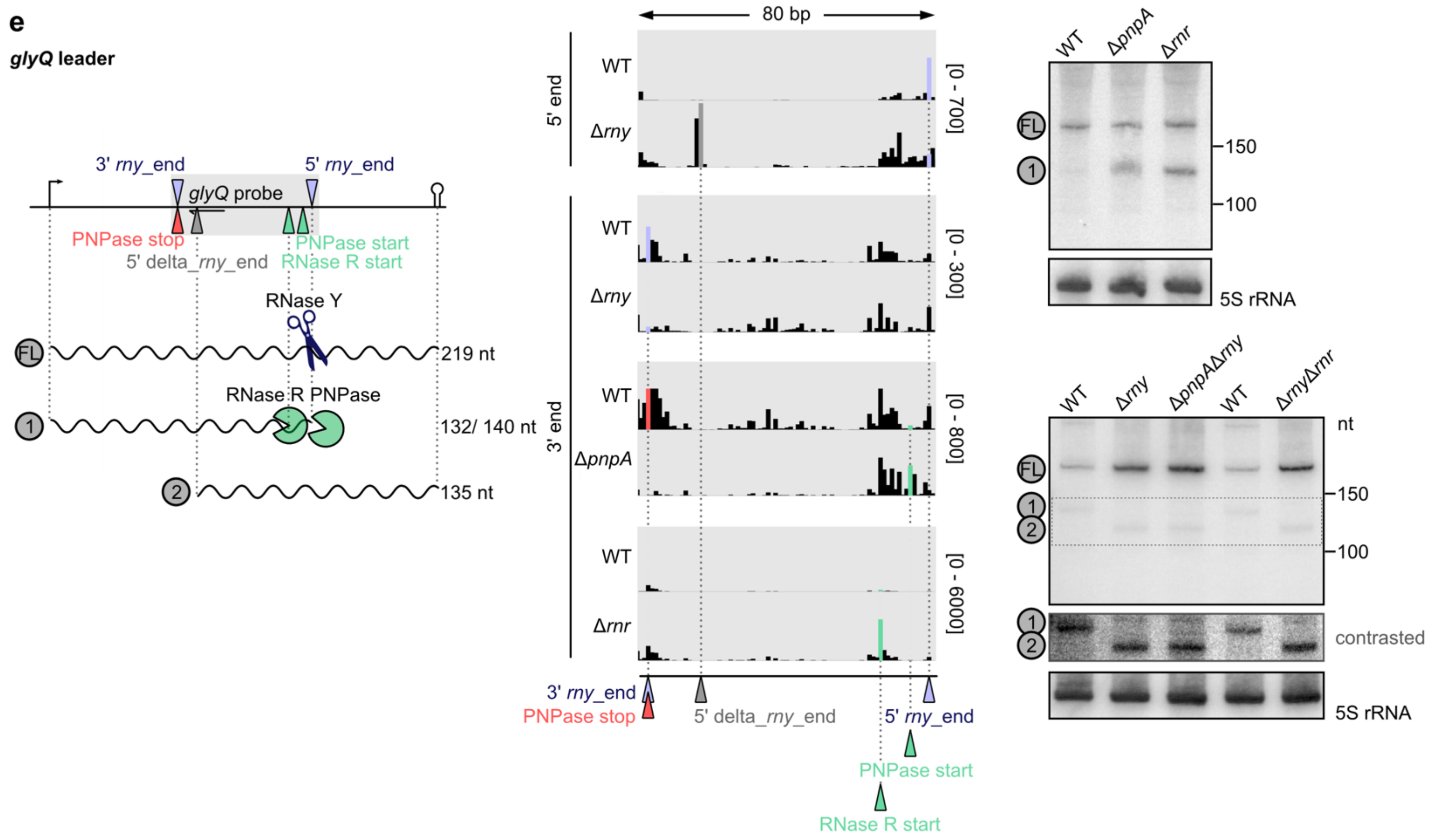

Supplementary Figure 7. RNase Y and PNPase are involved in the degradation of putative regulatory 5′ elements.

a–e Schematic loci representation (left) and 3′ end coverage profiles (for *glyQ* T-box also 5′ end coverage; middle) of putative 5′ regulatory elements (T-boxes or riboswitches), and corresponding Northern blot analyses (right). The coverage scale, which is equal for lanes within the grey rectangles, is shown between brackets. Predicted promoters, terminators, 3′-to-5′ exoRNase start and stop positions (green and red arrowheads, respectively), 3′ and 5′ *rny*_ends (purple arrowheads) are indicated. The RNA 5′ ends, which were more abundant in the Δ*rny* and Δ*pnpA* strains compared to the WT strain are named as 5′ delta_*rny*_end (grey arrowhead) and 5′ Δ*pnp*A_end (green arrowhead), respectively. The probes used in the Northern blot analyses and the expected sizes of the full length (FL) and decay intermediates are shown. The Northern blot analyses were performed in the WT, Δ*rny*, Δ*pnpA* and Δ*rny*Δ*pnpA* strains (for *glyQ* T-box also in the Δ*rnr* and Δ*rny*Δ*rnr* strains), and the 5S rRNA was used as a loading control. Shown are the results of one Northern blot analysis (n=3). For the poorly detectable RNAs, a contrasted portion of the blot is shown below the respective full blot. **a TPP.** RNase Y is responsible for the production of the decay intermediate 3′ end. The decay intermediate 5′ end (5′ end present in the Δ*pnpA* strain but not in the WT strain) was probably generated by an unidentified endoRNase[1]. **b, c *serS* and *valS*.** For the *serS* and *valS* T-boxes, we observed a 3′ *rny*_end that did not correspond to any PNPase trimming start position located in its proximity. However, by increasing the window size (from 5 to 1000 nt) to search for 3′-to-5′ exoRNase trimming start positions, we identified a PNPase start in both *serS* and *valS*, upstream of the identified 3′ *rny*_ends. These PNPase starts corresponded to the initial RNase Y processing positions. In the *serS* 5′ UTR, we detected an additional smaller decay intermediate in the WT strain. It is possible that PNPase initiated the

degradation of the RNase Y-generated decay intermediate and then stalled (putative PNPase stop position), leading to the production of this short isoform. Since the trimming of PNPase was RNase Y-dependent, the putative PNPase stop position was retrieved as a *rny*_3′ end. **d *thrS*.** PNPase was shown to be involved in the degradation of two decay intermediates in the *thrS* 5′ UTR[1]. Although we did not retrieve any *rny*_ends in this 5′ UTR, we observed by Northern blot analyses that these decay intermediates were generated by RNase Y. **e *glyQ*.** RNase Y processed the *glyQ* T-box, generating a decay intermediate that was fully degraded by RNase R or partially by PNPase up to the stop position. In the Northern blot analysis, we observed a smaller RNA in the Δ*rny* strain, which was likely produced by an endoRNase that replaced RNase Y in the *glyQ* leader. Consistent with this observation, an RNA 5′ end more abundant in the Δ*rny* strain than in the WT strain (5′ delta_*rny*_end) was retrieved in the *glyQ* 5′ UTR (Supplementary Data 1). Source data are provided as a Source Data file.

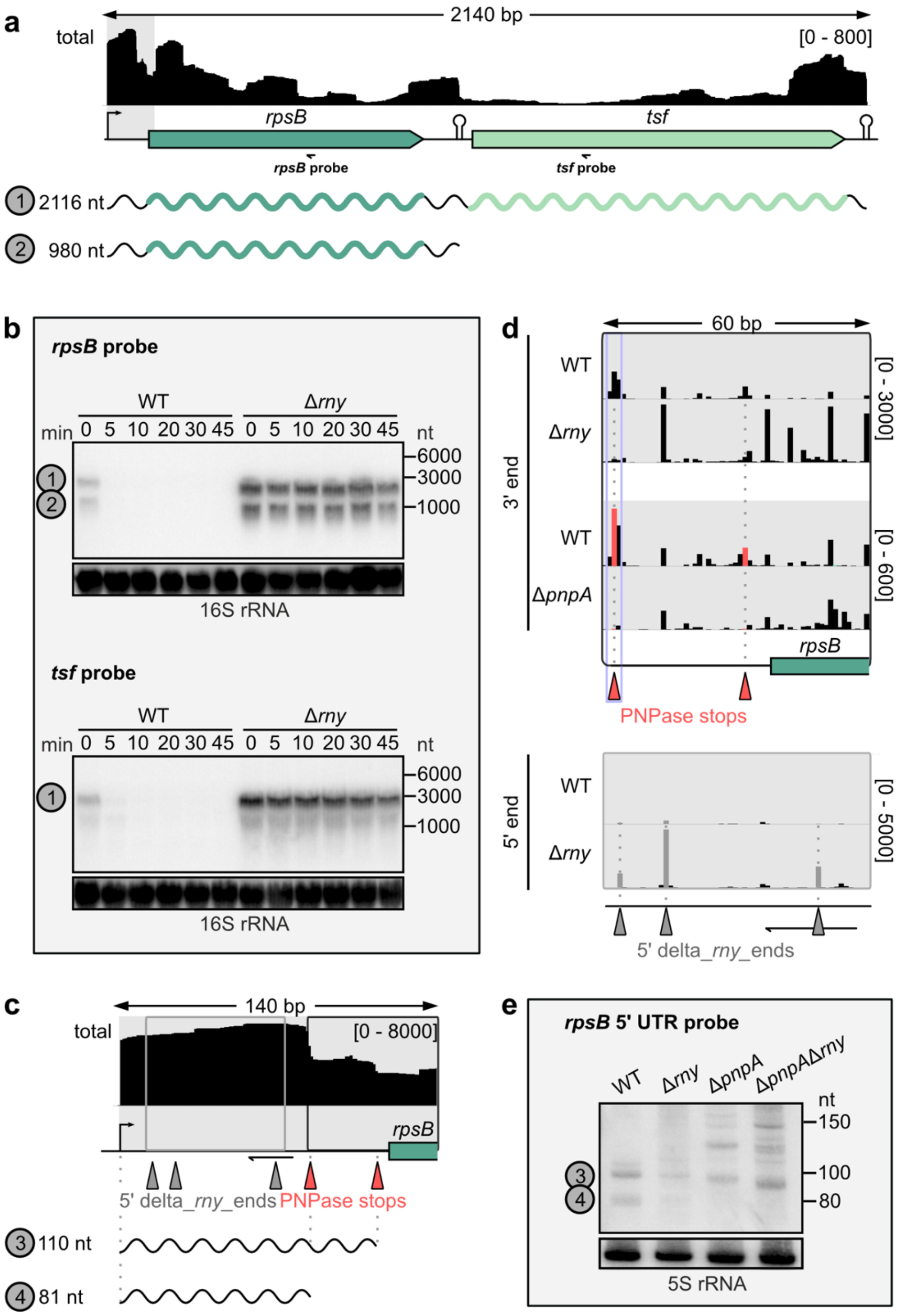

Supplementary Figure 8. RNase Y strongly affects the stability of the *rpsB* and *rpsB-tsf* transcripts.

a Total coverage profiles in the WT strain by RNA sequencing of transcripts of the *rpsB-tsf* operon and schematic representation of the locus. The coverage scale is indicated between brackets. The location of the promoter, terminator and probes used in the Northern blot analyses, and the predicted transcript sizes are shown. The grey rectangle highlights the region shown in panel c. *rpsB* codes for the highly conserved 30S ribosomal protein S2 that is essential for the translation process in all domains of life[2].

tsf encodes the elongation factor thermos-stable (EF-Ts), which allows guanine nucleotide exchange of the thermo unstable elongation factor (EF-Tu), itself responsible for transferring the aminoacyl-tRNA to the ribosome[3]. A predicted terminator in the intergenic region between *rpsB* and *tsf* ensures the production of the *rpsB* monocistronic isoform. *tsf* does not harbor its own promoter region, opposite to what was observed in *S. aureus*, where the *rpsB*-*tsf* intergenic region also contains a small ORF[4]. The deletion of RNase Y led to a significant increase of the *rpsB*-*tsf* operon abundance (~ 3-fold) (Supplementary Table 7). **b** The stability of *rpsB* and *rpsB*-*tsf* transcripts was determined by Northern blot analyses up to 45 minutes (min) of bacterial growth after the addition of rifampicin. 16S rRNA was used as a loading control. Shown are the results of one representative Northern blot analysis (n=3). RNase Y was already shown to dramatically affect the *rpsB*-*tsf* operon expression in *S. aureus* and to cleave the *rpsB* mRNA and the UTR between *rpsB* and *tsf* [4]. Here, we did not find any RNase Y cleavage sites in the *rpsB*-*tsf* operon (Supplementary Table 1). Therefore, although this operon is an RNase Y target in both *S. pyogenes* and *S. aureus*, the mechanism of regulation mediated by RNase Y is different in the two bacterial species. **c** Total coverage profile of *rpsB* 5′ UTR from RNA sequencing with annotation of the PNPase trimming stop positions and the RNA 5′ ends that were more abundant in the Δ*rny* strain than in the WT strain (5′ delta_*rny*_ends). The 5′ delta_*rny*_ends could be produced by an endoRNase that processes the *rpsB* 5′ UTR in the absence of RNase Y. The grey and black rectangles depict the regions of *rpsB* 5′ UTR that are shown in panel d. **d** 3′ and 5′ end RNA sequencing coverages of a region of *rpsB* 5′ UTR in the WT and Δ*rny* strains (for the 3′ end, also in the Δ*pnpA* strain). The coverage scale, which is equal for lanes within the grey rectangle, is shown between brackets. The PNPase trimming stop positions and the 5′ delta_*rny*_ends are depicted with red and grey arrowheads, respectively. The purple rectangles indicate the PNPase trimming stop positions that we hypothesized to be RNase Y-dependent. **e** Northern blot analysis of *rpsB* 5′ UTR in the WT, Δ*rny*, Δ*pnpA*, and Δ*rny*Δ*pnpA* strains. The probe used and the predicted transcript isoforms are shown in panel c. Shown are the results of one representative Northern blot analysis (n=3). The 5S rRNA was used as a loading control. The shorter isoform (number 4) was completely absent in both Δ*pnpA* and Δ*rny*Δ*pnpA* strains. Additional *rpsB* 5′ UTR truncated isoforms were detectable in the Δ*rny*, Δ*pnpA*, and Δ*rny*Δ*pnpA* strains, but not in the WT strain, indicating that they could be produced by the additional endoRNase that processes the *rpsB* 5′ UTR in the absence of RNase Y. Source data are provided as a Source Data file.

Strain	Relevant characteristics	Source
Streptococcus pyogenes		
**WT**<br>EC2224	SF370 (M1 serotype)	ATCC 700294 [2]
**Δ*rny***<br>EC2246	EC2224Δ*rny*::lox72	[7]
**Δ*rnr***<br>EC2254	EC2224Δ*rnr*::lox72	[1]
**Δ*pnpA***<br>EC2297	EC2224Δ*pnpA*::lox72	[1]
**Δ*rny*::*rny***<br>EC2298	EC2246Δlox72::*rny*-TT3-lox72	[8]
**Δ*yhaM***<br>EC2347	EC2224ΔSPy_0267::lox71- P*ermAM/B*-*ermAM/B*-lox66	[1]
**Δ*pnpA*Δ*rny***<br>EC2389	EC2297Δ*rny*::lox71- P*ermAM/B*-*ermAM/B*-lox66	[1]
**Δ*rny*Δ*rnr***<br>EC2310	EC2246Δ*rnr*::lox72	[1]
**Δ*yhaM*Δ*rny***<br>EC2392	EC2347Δ*rny*::lox71- P*ermAM/B*-*ermAM/B*-lox66	[1]

Oligo	Sequence 5′-3′ [a]	F/R [b]	Usage [c]	Target
**Oligos used for Northern blot analyses**				
OliRN243	*CGTTGTACCAACCATTGTAGC*	R	NB	16S rRNA
OLEC288	*CTAAGCGACTACCTTATCTCA*	R	NB	5S rRNA
OLEC8864	*TGGTCACAATCCCTACGCTT*	R	NB	TPP riboswitch
OLEC8857	*TCACAGCATCCACAGACTCT*	R	NB	*serS*
OLEC8860	*CGATTTCCGCGGTACCAC*	R	NB	SPy_1570-SPy_1569-*valS*
OLEC8856	*CTTTCCACCAACCAGCACTC*	R	NB	*thrS*
OLEC8866	*TTAATCACACTGCCCAAACGG*	R	NB	*glyQ*
OLEC5805	*TATCTTCGATACCGCCCAAG*	R	NB	*rpsB*
OLEC8344	*CTTCGACTTGCAGGCTAACC*	R	NB	*rpsB*
OLEC5807	*TCGTTGTTAGCTGGTTTGCC*	R	NB	*tsf*
OLEC5799	*ACAGCCACTGACGCTAAACC*	R	NB	*bmpA*
OLEC5797	*TCTGCCATTACCTGACGACA*	R	NB	*cdd*
OLEC10381	*TTGCGCTGCCTTCAATGAAT*	R	NB	SPy_1551
OLEC10384	*GATAGTGATGCCCGCTTGTT*	R	NB	*murC*
OLEC10501	*TGCCACCGTTCTTACCGTA*	R	NB	SPy_0316
OLEC8749	*CACGTTGAATATTGCCAGCTTCA*	R	NB	*pyrH*
OLEC8754	*CGACTGTCAGTGCTATTTCGCC*	R	NB	SPy_2197
**Oligos used for Primer extension analyses**				
OLEC4031	*CACTGACGCTAAACCAAGAC*	R	PE	*bmpA*

Supplementary Table 1. Strains, plasmids and oligos used in the study.

[a] *italic*: sequence annealing to the template.
[b] F: forward primer; R: reverse primer.
[c] NB: Northern blot; PE: primer extension.

Sample name	Total reads	Mapped reads	Uniquely mapped reads	Mapped reads after deduplication
WT_A	93780230	85606752	73716934	9034804
WT_B	92448016	85356859	76574989	5625154
WT_C	96370502	88752262	76921184	5273870
Δ*rny*_A	94163896	88084465	81507501	6593520
Δ*rny*_B	81619860	70437279	63616496	6424414
Δ*rny*_C	83093746	77014619	70684057	5772930
Δ*rny*::*rny*_A	63821288	55203447	47783051	4992048
Δ*rny*::*rny*_B	82868846	71770450	60537587	4232748
Δ*rny*::*rny*_C	95106882	83237055	75408001	2741284

Supplementary Table 2. Number of mapped reads.

Number of mapped reads for the WT, Δ*rny* and Δ*rny* complemented with the *rny* gene (Δ*rny::rny*) strains, for the three replicates (A, B and C).

SUPPLEMENTARY REFERENCES

1. Lécrivain, A.-L. *et al. In vivo* 3′-to-5′ exoribonuclease targetomes of *Streptococcus pyogenes*. *Proc. Natl. Acad. Sci. U.S.A.* **115**, 11814–11819 (2018).
2. Wilson, D. N. & Nierhaus, K. H. Ribosomal proteins in the spotlight. *Crit. Rev. Biochem. Mol. Biol.* **40**, 243–267 (2005).
3. Thirup, S. S., Van, L. B., Nielsen, T. K. & Knudsen, C. R. Structural outline of the detailed mechanism for elongation factor Ts-mediated guanine nucleotide exchange on elongation factor Tu. *J. Struct. Biol.* **191**, 10–21 (2015).
4. Khemici, V., Prados, J., Linder, P. & Redder, P. Decay-initiating endoribonucleolytic cleavage by RNase Y is kept under tight control via sequence preference and sub-cellular localisation. *PLoS Genet.* **11**, e1005577 (2015).
5. Suvorov, A. N. & Ferretti, J. J. Physical and genetic chromosomal map of an M type 1 strain of *Streptococcus pyogenes*. *J. Bacteriol.* **178**, 5546–5549 (1996).
6. Ferretti, J. J. *et al.* Complete genome sequence of an M1 strain of *Streptococcus pyogenes*. *Proc. Natl. Acad. Sci. U.S.A.* **98**, 4658–4663 (2001).
7. Le Rhun, A. *et al.* Identification of endoribonuclease specific cleavage positions reveals novel targets of RNase III in *Streptococcus pyogenes*. *Nucleic Acids Res.* **45**, 2329–2340 (2017).
8. Broglia, L. *et al.* RNase Y-mediated regulation of the streptococcal pyrogenic exotoxin B. *RNA Biol.* **15**, 1336–1347 (2018).
