# Supplementary material for: An RNA-seq based comparative approach reveals the transcriptome-wide interplay between 3′-to-5′ exoRNases and RNase Y
Source: Nat Commun. 2020 Mar 27;11:1587. doi: 10.1038/s41467-020-15387-6 (PMC7101322; doi:10.1038/s41467-020-15387-6)
Supplement: Supplementary file 3 — Reporting Summary [file 41467_2020_15387_MOESM3_ESM.pdf]

nature research

Corresponding author(s): Anaïs Le Rhun and Emmanuelle Charpentier

Last updated by author(s): Jan 21, 2020

Reporting Summary

Nature Research wishes to improve the reproducibility of the work that we publish. This form provides structure for consistency and transparency in reporting. For further information on Nature Research policies, see Authors & Referees and the Editorial Policy Checklist.

Statistics

For all statistical analyses, confirm that the following items are present in the figure legend, table legend, main text, or Methods section.

n/a	Confirmed	
☐	☒	The exact sample size (*n*) for each experimental group/condition, given as a discrete number and unit of measurement
☐	☒	A statement on whether measurements were taken from distinct samples or whether the same sample was measured repeatedly
☒	☐	The statistical test(s) used AND whether they are one- or two-sided<br>*Only common tests should be described solely by name; describe more complex techniques in the Methods section.*
☒	☐	A description of all covariates tested
☒	☐	A description of any assumptions or corrections, such as tests of normality and adjustment for multiple comparisons
☐	☒	A full description of the statistical parameters including central tendency (e.g. means) or other basic estimates (e.g. regression coefficient) AND variation (e.g. standard deviation) or associated estimates of uncertainty (e.g. confidence intervals)
☐	☒	For null hypothesis testing, the test statistic (e.g. *F*, *t*, *r*) with confidence intervals, effect sizes, degrees of freedom and *P* value noted<br>*Give P values as exact values whenever suitable.*
☒	☐	For Bayesian analysis, information on the choice of priors and Markov chain Monte Carlo settings
☒	☐	For hierarchical and complex designs, identification of the appropriate level for tests and full reporting of outcomes
☒	☐	Estimates of effect sizes (e.g. Cohen's *d*, Pearson's *r*), indicating how they were calculated

Our web collection on statistics for biologists contains articles on many of the points above.

Software and code

Policy information about availability of computer code

Data collection	The Northern blot and primer extension analysis data were collected and visualized by using Typhoon TM FLA9500 (GE Healthcare) using the control software (version 1.1) and the image processing package Fiji (available at https://imagej.net/Fiji), respectively. RNA sequencing raw data were collected on Illumina HiSeq3000 platform at the Max Planck-Genome-centre Cologne. The data were visualized using Integrative Genomics Viewer (IGV_2.4.14) (available at https://software.broadinstitute.org/software/igv/).
Data analysis	After the quality filter (using FastQC v0.11.5, available at https://www.bioinformatics.babraham.ac.uk/projects/fastqc/) and adapter sequence removal (using Cutadapt v1.11 available at https://cutadapt.readthedocs.io/en/v1.11/index.html), RNA sequencing reads were mapped to the reference S. pyogenes genome (NC_002737.2) (using STAR v2.5.2b, available at https://code.google.com/archive/p/rna-star/). Gene abundance (counts) was determined using featureCounts (v1.5.2) and differentially expressed genes were identified uding edgeR (v3.20.6) (both available at http://www.bioconductor.org.).<br>The RNase processing sites were identified using a previously published method based on edgeR (v3.20.6) and custom parameters (Le Rhun, Lécrivain et al., 2016 Nucleic Acids Res. and Lécrivain, Le Rhun et al., 2018, Proc. Natl. Acad. Sci. U.S.A.). Python (v3.6.3) was used to compare the location of the RNase processing sites. RNAfold (v2.4.3) (available at https://www.tbi.univie.ac.at/RNA/) was used to calculate the minimum free energy. WebLogolib (v3.5.0) (available at http://weblogo.threeplusone.com) was used to generate the sequence logos.

For manuscripts utilizing custom algorithms or software that are central to the research but not yet described in published literature, software must be made available to editors/reviewers. We strongly encourage code deposition in a community repository (e.g. GitHub). See the Nature Research guidelines for submitting code & software for further information.

Data

Policy information about availability of data

All manuscripts must include a data availability statement. This statement should provide the following information, where applicable:

- Accession codes, unique identifiers, or web links for publicly available datasets
- A list of figures that have associated raw data
- A description of any restrictions on data availability

The RNA sequencing data from the wild type and RNase Y deletion mutant and complemented RNase Y deletion mutant strains, generated in this study, have been deposited at the National Center for Biotechnology Information (NCBI) under the accession number SRP149896. The RNA sequencing data from the different exoRNase deletion mutants, which were used in this study, are available at NCBI under the accession number SRP149887.
The source data underlying the Figures 5c, 6c, 7b, 8c, d, e and Supplementary Figures 4, 5c–e, 6, 7 are provided as Source Data file

Field-specific reporting

Please select the one below that is the best fit for your research. If you are not sure, read the appropriate sections before making your selection.

☒ Life sciences ☐ Behavioural & social sciences ☐ Ecological, evolutionary & environmental sciences

For a reference copy of the document with all sections, see nature.com/documents/nr-reporting-summary-flat.pdf

Life sciences study design

All studies must disclose on these points even when the disclosure is negative.

Sample size	RNA sequencing, for each condition, was repeated in three biological replicates and the Northern blot analyses and Primer extension analyses were performed at least in three biological replicates.
Data exclusions	FastQC (v0.11.5) was used to assess the quality of the RNA sequencing data and poor quality reads (with quality score <10) were filtered. Reads smaller than 18 nt were discarded. To search for RNase processing sites, the genome coverage was filtered with counts per million (cmp) value ≥ 0.05. Only RNA ends with cmp ≥ 5 were further analyzed, as described in Lécrivain, Le Rhun et al., 2018, Proc. Natl. Acad. Sci. U.S.A.
Replication	All results described in this manuscript were reliably reproduced.
Randomization	Not applicable
Blinding	Not applicable

Reporting for specific materials, systems and methods

We require information from authors about some types of materials, experimental systems and methods used in many studies. Here, indicate whether each material, system or method listed is relevant to your study. If you are not sure if a list item applies to your research, read the appropriate section before selecting a response.

Materials & experimental systems

n/a	Involved in the study	
☒	☐	Antibodies
☒	☐	Eukaryotic cell lines
☒	☐	Palaeontology
☒	☐	Animals and other organisms
☒	☐	Human research participants
☒	☐	Clinical data

Methods

n/a	Involved in the study	
☒	☐	ChIP-seq
☒	☐	Flow cytometry
☒	☐	MRI-based neuroimaging
