# Supplementary material for: An RNA-seq based comparative approach reveals the transcriptome-wide interplay between 3′-to-5′ exoRNases and RNase Y
Source: Nat Commun. 2020 Mar 27;11:1587. doi: 10.1038/s41467-020-15387-6 (PMC7101322; doi:10.1038/s41467-020-15387-6)
Supplement: Supplementary file 4 — Description of Additional Supplementary Files [file 41467_2020_15387_MOESM4_ESM.pdf]

Description of Additional Supplementary Files

File Name: Supplementary Data 1
Description: List of RNA ends present in the WT strain (rny_ends) or present in the Δrny strain (delta_rny_ends). List of RNA ends more abundant in the WT strain (“rny_ends” tab) or in the Δrny strain (“delta_rny_ends” tab). Information is given about the chromosome position; the strand: positive (+) or negative (−); the RNA end (5′ or 3′); the classification of RNA ends: unique (U) or stepped (S); the log 2 fold change (Log 2 FC); the false discovery rate (FDR); the proportion of ends; the ratio of proportion of ends; the gene ID (“<->” indicates the intergenic region between two genes); the description of the gene function as reported in NCBI; the location of the end: open reading frame (ORF) or untranslated region (UTR); the nt upstream of RNase Y processing (nt in 3′); the distance in nt to the closest predicted terminator and the ID number of the terminator.

File Name: Supplementary Data 2
Description: List of 3′ rny_ends corresponding to 3′-to-5′ exoRNase trimming stop positions. The identified 3′ rny_ends were compared to the trimming stop positions of PNPase, YhaM and RNase R (i.e. RNA ends more abundant in the WT than in ΔpnpA, ΔyhaM, and Δrnr strains, respectively) (see Methods). Information is provided about the chromosome position, the strand: positive (+) or negative (−), the gene ID (“<->”: intergenic region between two genes), the description of the gene function as reported in NCBI and the location: open reading frame (ORF) or untranslated region (UTR).

File Name: Supplementary Data 3
Description: Retrieval of the initial RNase Y processing positions. To identify the initial processing position of RNase Y (see Methods), the identified 3′ rny_ends corresponding to 3′-to-5′ exoRNase stop positions (see Supplementary Data 2) were compared with the exoRNase trimming start positions located downstream. Information is provided about the chromosome position, the strand: positive (+) or negative (−); the distance (nt) between the two positions (processivity); the gene ID (“<->”: intergenic region between two genes); the gene description as reported in NCBI; the location: open reading frame (ORF) or untranslated region (UTR); the nt of the exoRNase trimming start position and for PNPase targets, the 3 nts following the trimming start position are also reported.

File Name: Supplementary Data 4
Description: List of 3′ rny_ends corresponding to 3′-to-5′ exoRNase trimming start positions. The identified 3′ rny_ends (excluding those from Supplementary Data 2) were compared with the trimming start positions of PNPase, YhaM and RNase R (i.e. RNA ends more abundant in the ΔpnpA, ΔyhaM and Δrnr strains than in the WT strain, respectively) (see Methods). Information is provided about the chromosome position, the strand: positive (+) or negative (−), the distance between the two RNA 3′ ends, the gene ID (“<->”: intergenic region between two genes), the description of the gene function as reported in NCBI and the identity of the nt at the exoRNase trimming start position.

File Name: Supplementary Data 5

Description: List of PNPase trimming start positions located close to 5′ rny_ends. RNA 5′ ends generated by RNase Y were associated with PNPase trimming start positions (see Methods). Information is given about the chromosome position; the strand: positive (+) or negative; the distance in nt between the two RNA ends; the gene ID (“<->” indicates the intergenic region between two genes); the description of the gene function as reported in NCBI; the sequence around PNPase trimming start position (capital letter) and the RNA 5′ end produced by RNase Y processing (red letter).

File Name: Supplementary Data 6
Description: List of RNA fragments produced by RNase Y. List of 3′ rny_ends and 5′ rny_ends that were located 50 to 200 nt from each other. Information is provided about the chromosome position; the nt upstream of the 5′ rny_ends (nt upstream) and the nt at the 3′ rny_ends (nt at 3′); the distance (nt) between the paired 3′ rny_end and the 5′ rny_end; the strand: positive (+) or negative (−); the location: open reading frame (ORF) or untranslated region (UTR); the chromosome positions of the PNPase or YhaM trimming stops when matched with the 3′ rny_ends and the positions of the respective PNPase or YhaM starts if identified; the gene ID (“<->”: intergenic region between two genes) and the description of the gene function as reported in NCBI).

File Name: Supplementary Data 7
Description: Genes and terminators differentially expressed in the Δrny strain. The genes located in a same transcript are grouped with black lines. The grey genes are encoded in the prophage SF370.1. Information is given about the gene ID, the gene name, the log 2 Fold Change (Log 2 FC) and the False Discovery Rate (FDR). The “X” indicates that a processing by RNase Y (WT 5′ or 3′ end) was retrieved in the RNAs or terminators.
